# Intraoperative early change of latencies in Facial nerve function during Microvascular Decompression studied in patients with Hemifacial Spasm

**Published:** 2012-11

**Authors:** Mohsen Dalvandi, Alireza Jamshidi Fard, Alireza Mohammdi

**Affiliations:** ^*a*^Department of Neurosurgery, School of Medicine, Arak University of Medical Sciences, Arak, Iran.

## Abstract

**Background:**

Primary hemifacial spasm (HFS) is reported mainly as a result of cross compression of blood vessel and facial nerve at its root exit zone (REZ). Generation of HFS could be due to hyperexcitability of facial nerve since microvascular decompression (MVD) has been an effective treatment in clinical experince of authors. Multimodal Intraoperative monitoring (MIOM) has been frequently used for care and constant evaluation of facial nerve during MVD. In HFS patients F waves of the facial muscle which is known to be induced from backfiring of facial motor nucleus, Blink reflexes and Lateral spread (abnormal muscle responses) before, during and after MVD have been used to evaluate the excitability of the facial motor nucleus and the treatment outcome after MVD procedure.

**Methods:**

In 9 female HFS cases with abnormal lateral spread in their facial Electromyography (EMG), patients history, preoperative physical examination, electrodiagnosis and MIOM were perfored using a Moltimodal 40 channels electrophysiologic monitoring system (Nicolet Endeavor, VIASYS Healthcare, 2005, USA). Free run EMG, Stimulated EMG, bilateral blink reflexes and facial F waves were set for MIOM as indicators of MVD effectiveness.

Bilateral Orbicularis Oris/Oculi muscles were used for surface EMG recordings. Antidromic stimulatin of facial nerve branches and direct bipolar stimulation of the nerve in REZ, before and after its vascular contact at the site of operation applied by surgen. In all subjects, recordings were scheduled a week before, intaoperatively and every 2 weeks up to 3 months after operation.

**Results:**

In affected side of the face before MVD, threshold of F wave was reduced and excitability of blink reflex EMG responses were enhanced compared to the normal side. These responses remaind the same intraoperatively. In post operation recordings F waves and Blink reflex responses were as before in 7 subjects up to fourth week after the surgery, although with lower amplitude of the responses. These responses and Lateral spread of EMGs disappeared completely after 6 weeks. In 2 other subject these abnormal findings reduced slowly and subsequently disappeared after 12 weeks. In all cases, introperative recordings of F wave latencies and the latencies of R1 and R2 responses of blink reflexes were reduced significantly even before 20 minutes of decompression (P less than 0.03).

Propofol or Propofol/Ketamine mixture plus narcotic is suitable to obtain stable reproducible F waves and EMGs. Atracurium or other nondepolarizing muscle relaxant should be avoided. Muscle relaxants, mean arterial pressure (MAP) below 70 mmHg may cause bilateral reduction or loss of reflex responces and EMGs. In all cases, there was no postoperative clinically detectable complication.

**Conclusions:**

MIOM could be useful technique in all patients undergoing any procedures around cranial nerves. Monitoring can practically reduce possibilities of neurologic deficit and reduce the potential risk of interventions around facial REZ. We conclude that in MVD for HFS, the use of stimulated EMGs for evaluating involved facial nerve is not sufficent because, practically, it could be limited to the efferent nerve fibers not the nucleuses.In these settings and similar procedures, if monitoring systems are avaiable, alternative multimodal methods with greater sensitivity and efficacy should be explored. MVD seems to be effective procedure for treatment of HFS and monitoring would help to optimise the MVD.

The study also supports the hypothesis that the hyperexcitability of the facial motor nucleus may be the main cause of hemifacial spasm. To aquire and maintain MIOM modalities, close collaboration of the anesthesioloist is nessesary.

**Keywords:**

Hemifacial spasm, Microvascular decompression, Multimodal intraoperative monitoring


					Volume 4, Suppl. 1 Nov 2012
Publisher: Kermanshah University of Medical Sciences
URL: http://www.jivresearch.org
Copyright:
Congress of Iranian Neurosurgeons, 14-16 November 2012, Kermanshah, Iran
Paper No. 89
Intraoperative early change of latencies in Facial nerve
function during Microvascular Decompression studied in
patients with Hemifacial Spasm
Mohsen Dalvandi a
, Alireza Jamshidi Fard a,*
, Alireza Mohammdi a
a
Department of Neurosurgery, School of Medicine, Arak University of Medical Sciences, Arak, Iran.
Abstract:
Background: Primary hemifacial spasm (HFS) is reported mainly as a result of cross compression of blood vessel and
facial nerve at its root exit zone (REZ). Generation of HFS could be due to hyperexcitability of facial nerve since
microvascular decompression (MVD) has been an effective treatment in clinical experince of authors. Multimodal
Intraoperative monitoring (MIOM) has been frequently used for care and constant evaluation of facial nerve during
MVD. In HFS patients F waves of the facial muscle which is known to be induced from backfiring of facial motor
nucleus, Blink reflexes and Lateral spread (abnormal muscle responses) before, during and after MVD have been
used to evaluate the excitability of the facial motor nucleus and the treatment outcome after MVD procedure.
Methods: In 9 female HFS cases with abnormal lateral spread in their facial Electromyography (EMG), patients
history, preoperative physical examination, electrodiagnosis and MIOM were perfored using a Moltimodal 40
channels electrophysiologic monitoring system (Nicolet Endeavor, VIASYS Healthcare, 2005, USA). Free run EMG,
Stimulated EMG, bilateral blink reflexes and facial F waves were set for MIOM as indicators of MVD effectiveness.
Bilateral Orbicularis Oris/Oculi muscles were used for surface EMG recordings. Antidromic stimulatin of facial nerve
branches and direct bipolar stimulation of the nerve in REZ, before and after its vascular contact at the site of
operation applied by surgen. In all subjects, recordings were scheduled a week before, intaoperatively and every 2
weeks up to 3 months after operation.
Results: In affected side of the face before MVD, threshold of F wave was reduced and excitability of blink reflex
EMG responses were enhanced compared to the normal side. These responses remaind the same intraoperatively. In
post operation recordings F waves and Blink reflex responses were as before in 7 subjects up to fourth week after
the surgery, although with lower amplitude of the responses. These responses and Lateral spread of EMGs
disappeared completely after 6 weeks. In 2 other subject these abnormal findings reduced slowly and subsequently
disappeared after 12 weeks. In all cases, introperative recordings of F wave latencies and the latencies of R1 and
R2 responses of blink reflexes were reduced significantly even before 20 minutes of decompression (P less than
0.03).
Propofol or Propofol/Ketamine mixture plus narcotic is suitable to obtain stable reproducible F waves and EMGs.
Atracurium or other nondepolarizing muscle relaxant should be avoided. Muscle relaxants, mean arterial pressure
(MAP) below 70 mmHg may cause bilateral reduction or loss of reflex responces and EMGs. In all cases, there was
no postoperative clinically detectable complication.
Conclusions: MIOM could be useful technique in all patients undergoing any procedures around cranial nerves.
Monitoring can practically reduce possibilities of neurologic deficit and reduce the potential risk of interventions
around facial REZ. We conclude that in MVD for HFS, the use of stimulated EMGs for evaluating involved facial
nerve is not sufficent because, practically, it could be limited to the efferent nerve fibers not the nucleuses.In these
settings and similar procedures, if monitoring systems are avaiable, alternative multimodal methods with greater
sensitivity and efficacy should be explored. MVD seems to be effective procedure for treatment of HFS and
monitoring would help to optimise the MVD.
Volume 4, Suppl. 1 Nov 2012
Publisher: Kermanshah University of Medical Sciences
URL: http://www.jivresearch.org
Copyright:
Congress of Iranian Neurosurgeons, 14-16 November 2012, Kermanshah, Iran
The study also supports the hypothesis that the hyperexcitability of the facial motor nucleus may be the main cause of
hemifacial spasm. To aquire and maintain MIOM modalities, close collaboration of the anesthesioloist is nessesary.
Key words:
Hemifacial spasm, Microvascular decompression, Multimodal intraoperative monitoring
* Corresponding Author at:
Alireza Jamshidifard: School of Medicine, Arak University of Medical Science, Arak, Iran. Tel: +98 9181616251,
Email: arjfard@yahoo.com, (Jamshidifard A.).

				

